# Microbiota-Associated Therapy for Non-Alcoholic Steatohepatitis-Induced Liver Cancer: A Review

**DOI:** 10.3390/ijms21175999

**Published:** 2020-08-20

**Authors:** Yi-Hsun Chen, Wei-Kai Wu, Ming-Shiang Wu

**Affiliations:** 1 College of Medicine, National Taiwan University, Taipei 100, Taiwan; u9423201@gmail.com; 2 Division of Gastroenterology, National Taiwan University Hospital Bei-Hu Branch, Taipei 108, Taiwan; weikaiwu0115@gmail.com; 3 Department of Internal Medicine, National Taiwan University Hospital, Taipei 100, Taiwan

**Keywords:** gut microbiota, NAFLD, HCC, dysbiosis, metabolites

## Abstract

Even though advancement in medicine has contributed to the control of many diseases to date, cancer therapy continues to pose several challenges. Hepatocellular carcinoma (HCC) etiology is multifactorial. Recently, non-alcoholic fatty liver disease (NAFLD) has been considered as an important risk factor of HCC. NAFLD can be divided into non-alcoholic simple fatty liver (NAFL) and non-alcoholic steatohepatitis (NASH) based on histopathological features. Recently, studies have indicated that the gut microbiota is associated with NAFLD and HCC. Therefore, in this review, we have discussed the effects of gut microbiota-related mechanisms, including dysbiosis and gut barrier function, and gut microbiota-derived metabolites on NAFLD and HCC pathogenesis and the potential therapeutic strategies for NAFLD and HCC. With a better understanding of the gut microbiota composition and function, new and improved diagnostic, prognostic, and therapeutic strategies for common liver diseases can be developed.

## 1. Introduction

With advancements in medicine, many diseases can be controlled, but cancer continues to pose many challenges. Hepatocellular carcinoma (HCC) ranks fifth and second in highest cancer incidence and global cancer-related mortality, respectively [1,2]. Traditionally, chronic viral hepatitis caused by hepatitis B and C viruses (HBV and HCV, respectively) is a major risk factor of HCC [3]. In recent decades, the chronic viral hepatitis disease burden has been gradually controlled by universal implementation of HBV vaccination and dramatic improvement in anti-HBV and anti-HCV treatments [4]. Therefore, the incidence of viral hepatitis-related liver cirrhosis and HCC is expected to decline. Other important causative risk factors of HCC include non-alcoholic fatty liver disease (NAFLD), obesity, diabetes, and alcoholism [5]. Currently, NAFLD is the second most common cause of end-stage liver disease or liver cancer, which requires liver transplantation, in the United States [6,7,8]. It has been estimated that, from 2016 to 2030, the number of NAFLD-induced end-stage liver disease cases and related deaths worldwide will be doubled [9]. Therefore, NAFLD is likely to become the most important cause of HCC in the future.

**Figure 1:**
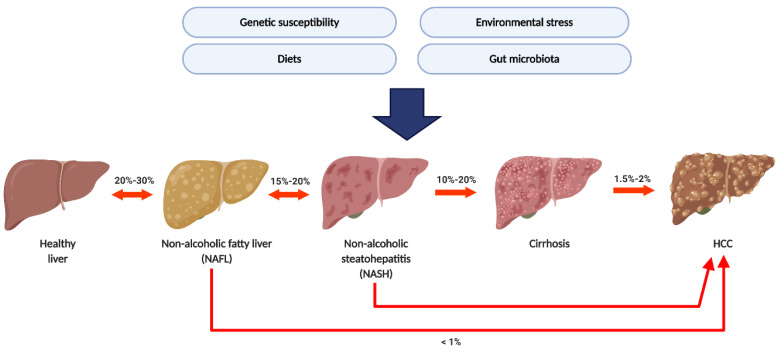
**The natural history of non-alcoholic fatty liver disease (NAFLD) and its etiological risk factor.** The prevalence of NAFL was around 20–30%. Around 15–20% of non-alcoholic simple fatty liver (NAFL) patients developed non-alcoholic steatohepatitis (NASH); 10–20% of NASH patients will further process to cirrhosis and Hepatocellular carcinoma (HCC). Several factors including genetic susceptibility, environmental stress, diet, and gut microbiota were considered as etiological risk factors.

Generally, NAFLD progression can be divided into four pathological stages, including non-alcoholic simple fatty liver (NAFL), non-alcoholic steatohepatitis (NASH), hepatic cirrhosis, and HCC [10] (Figure 1). Recent epidemiological studies showed that the average prevalence rate of NAFLD appeared to be higher in Asia (27%) than in the United States (24%) and Europe (23%) [7,8,11]. NAFLD-related health issues have attracted global attention since NAFLD is increasingly becoming the most common cause of hepatic cirrhosis and HCC [9,12]. NAFLD patients who remain in the NAFL stage show a much lower risk of developing HCC than NASH patients. Chronic and repetitive hepatocyte damage and repair in steatohepatitis may lead to advanced fibrosis, cirrhosis, and HCC, thus causing mortality within a few decades. Moreover, no FDA approved medication for NAFLD treatment exists presently. Therefore, there is an urgent and unmet need for a better understanding of NAFLD pathogenesis and progression to HCC.

Owing to this anatomical and functional connection, the liver and intestines maintain close functional and bidirectional communication which is subsumed in the “gut–liver axis”. The liver is continually exposed to the products of digestion, absorption, and gut-derived factors through the portal vein [13]. On the other hand, the liver plays an important role in the regulation of the gut microbiota composition via bile acids (BAs) [14]. Recent studies have suggested that dysbiosis, the change in the gut microbiota, may be associated with liver diseases, including NAFLD and HCC [15,16,17]. Increased intestinal permeability, which is associated with dysbiosis, leads to the liver being exposed to intestinal toxic factors and bacteria such as lipopolysaccharides (LPS) [18,19]. Exposure of liver with these factors further induced hepatic inflammation and damage which contributed to HCC pathogenesis. In this review, we have discussed the pathophysiological roles of the gut microbiota and relevant molecules in NAFLD progression to HCC. Some potential diagnostic and therapeutic highlights, which could be implemented for future clinical applications, were included.

## 2. From NAFL to NASH: The Gut Microbiota-Associated Mechanisms

Although NAFL patients bear lower risk of developing HCC than NASH patients, approximately 10–20% of NAFL patients progress to NASH, with a significantly increasing risk of developing cirrhosis and HCC. Therefore, understanding the mechanistic roles of the gut microbiota that trigger the NAFLD inflammatory status may potentially help in discovering novel microbial and molecular pathways for preventing HCC development.

### 2.1. NASH and Dysbiosis

Many studies have indicated that gut dysbiosis is associated with NAFLD pathogenesis [20,21,22,23,24,25,26]. Compared to healthy individuals, NASH patients exhibit increased relative abundance of *Blautia*, *Dorea*, *Lactobacillus*, *Clostridium*, *Allisonella*, *Parabacteroides*, and *Escherichia* spp. [20,21,25,26,27] and decreased relative abundance of *Oscillospira*, *Coprococcus*, *Faecalibacterium*, and *Bifidobacterium* spp. [20,25,26,27]. A common finding in NASH patients, compared to NAFL patients, is increased relative abundance of *Blautia* and *Bacteroides* spp. [23,28] and decreased relative abundance of *Prevotella* spp. [28]. Furthermore, gut dysbiosis includes not only compositional changes but also metabolic functional changes in the gut microbiome. For example, compositional changes in the gut microbiota lead to an altered short chain fatty acid (SCFA) profile, further affecting host energy absorption [20,25,29]. Gut dysbiosis can result in an increase in gut permeability, disruption of metabolic homeostasis, and changes in the microbiota-associated metabolites, thus eventually contributing to disease initiation and progression.

### 2.2. NASH and Leaky Gut

**Figure 2:**
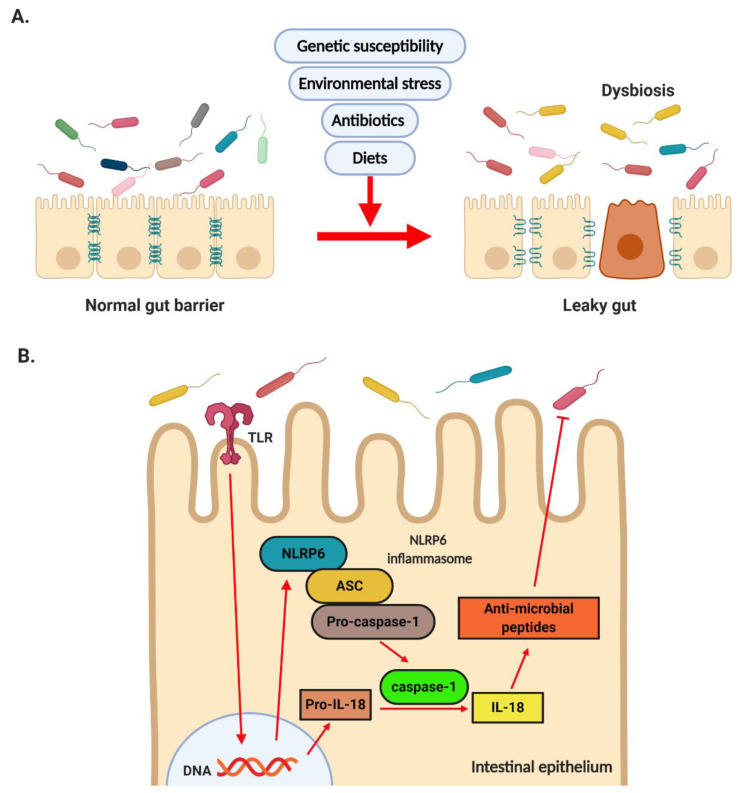
**Dysbiosis and the role of NLRP6.** (**A**) Dysbiosis induced by genetic susceptibility, environmental stress, diet, and gut microbiota results in disrupted tight junction and increased intestinal permeability. (**B**) Microbiota-derived factors activate NLRP6 inflammation via TLR signaling. Activation of NLRP6 results in induction of anti-microbial peptide synthesis and contributes to maintaining the homeostasis of gut microbiota.

In previous clinical studies, NASH patients exhibited greater intestinal permeability than simple steatosis patients and healthy individuals [30]. Increased intestinal permeability is caused by decreased expression of zonula occludens-1 (ZO-1), a representative tight junction protein [30,31,32]. Several bacteria, including *Lactobacillus*, *Bifidobacterium*, Bacteroidetes, *Clostridiales*, *Oscillibacter*, *Desulfovibrio* spp., and *Akkermansia muciniphila*, have been associated with intestinal permeability in animal models. Among these bacteria, *Lactobacillus*, *Bifidobacterium*, Bacteroidetes, *Clostridiales* spp., and *Akkermansia muciniphila* are considered gut barrier-promoting microbes, while *Oscillibacter* and *Desulfovibrio* spp. are considered gut barrier-disrupting microbes [33]. *Lactobacillus*, *Bifidobacterium* spp. and *A. muciniphila* induce ZO-1 expression to promote the gut barrier [34,35,36]. On the other hand, *Desulfovibrio* spp. produce genotoxic hydrogen sulfide (H_2_S), increasing the intestinal permeability [37]. Bacteroidetes, Verrucomicrobia, *Akkermansia*, and *Lactobacillus* spp. were positively correlated with increased expression levels of tight junction proteins, including ZO-1, occludin, and claudin-1, indicating that these bacteria maintained gut barrier function and improved hepatic inflammation and oxidative stress. On the other hand, Firmicutes, Proteobacteria, *Butyricimonas*, *Parabacteroides*, and *Bilophila* spp. exhibited the opposite effect [38]. Modulation of the gut microbiota by *Bifidobacterium bifidum* ATCC 2952 restored dextran sodium sulfate (DSS)-induced dysbiosis and up-regulated the expression of anti-inflammatory cytokines including interleukin (IL)-10, peroxisome proliferator-activated receptor (PPAR)-γ, and IL-6 in the gut, thereby indicating the important role of the gut microbiota [39]. A recent study, in which fecal microbial transplantation (FMT) from mice on high-fat diets (HFDs) to mice on standard diets was performed, showed the gut barrier damages in these mice, thereby indicating that altered gut microbiota was responsible for increased intestinal permeability [40] (Figure 2A).

Activation of the inflammasome by diverse microbial-, stress-, and danger-associated signals triggers pro-inflammatory cytokines including IL-1β and IL-18, thereby promoting innate immunity [41]. Previous studies have demonstrated that the intestinal epithelial nucleotide-binding oligomerization domain (NOD)-like receptor (NLR) family pyrin domain containing 6 (NLRP6) inflammasome maintains the intestinal barrier and microbial balance by regulating goblet cell mucus secretion [42] and anti-microbial peptide production [43]. NLRP6 is highly expressed in the epithelial cells of the small intestine, colon, and goblet cells and is co-expressed with apoptosis-associated speck-like protein containing a caspase recruitment domain (ASC) and caspase-1 in the intestinal epithelium [43]. A previous study indicated that fructose-fed mice exhibited impaired gut barrier and NLRP6 inflammasome [44]. NLRP6 activation induced the synthesis of anti-microbial peptides, including angiogenin-4, intelectin-1, and resistin-like molecule β, by gut epithelial cells [43]. Furthermore, NLRP6-deficient mice exhibited impaired anti-microbial peptides, resulting in dysbiosis, as indicated by the increased relative abundance of the *Prevotellaceae* spp. and members of the TM7 phylum and the decreased relative abundance of the *Lactobacillus* spp. and members of the Firmicutes phylum [45]. Therefore, the gut microbiota–NLRP6 axis plays an important role in maintaining the gut barrier function (Figure 2B).

### 2.3. Gut Microbiota and Hepatic Inflammation

**Figure 3:**
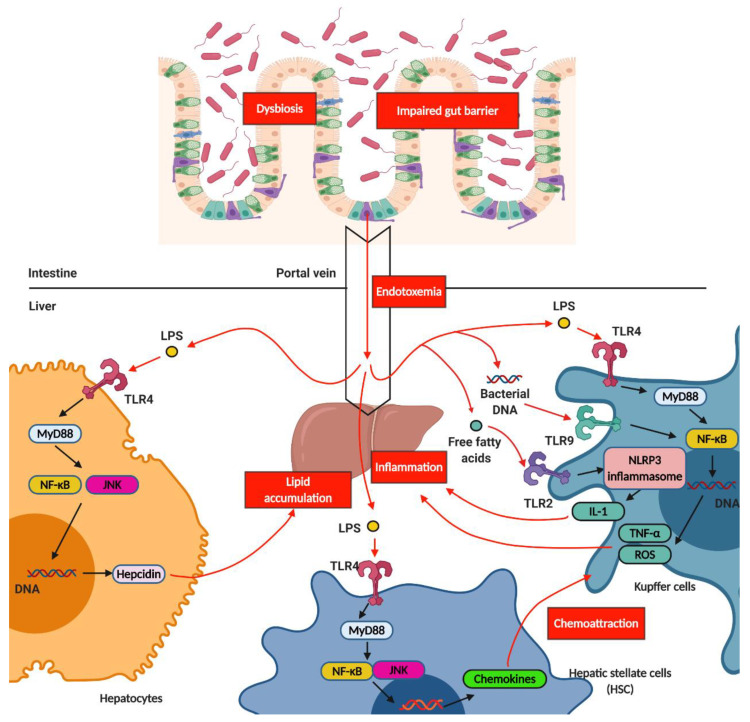
**The mechanism of hepatic inflammation induced by gut microbiota.** Dysbiosis results in impaired gut barrier and further induces the influx of bacterial DNA and LPS, which is termed endotoxemia, from gut to liver through portal vein. LPS further triggers TLR4 signaling in hepatocytes, Kupffer cells, and hepatic stellate cells (HSC). Activation of TLR4 and TLR9 in Kupffer cells induces the production of TNF-α and ROS, which further contributes to hepatic inflammation. Activation of TLR4 in hepatocytes induces the production of hepcidin, which further induces hepatic lipid accumulation. Activation of TLR4 in HSC induces the production of chemokines, which further contributes to chemoattraction for Kupffer cells. Additionally, influx of free fatty acid from gut to liver activates TLR2 signaling, which is termed lipotoxicity. Activation of TLR2 signaling results in activation of NLRP3 inflammation, which induces the production of IL-1. Increased IL-1 production leads to hepatic inflammation. LPS, Lipopolysaccharide; MyD88, myeloid differentiation primary response 88; NF-κB, nuclear factor kappa-light-chain-enhancer of activated B cells; JNK, c-Jun N-terminal kinase; TLR, toll-like receptors.

The gut microbiota signals travel through the human body systemically via the liver. Both nutrients and microbe-derived molecules from the intestinal lumen converge in the liver through the portal vein. Modulation of intestinal permeability regulates the entry of microbe-derived molecules into the liver from the gut. Some of these molecules are harmful substances that can cause liver inflammation and induce the pathological process of NASH. For example, in JAM-A-deficient mice (genetically induced gut barrier dysfunction model) and a DSS-induced gut inflammation animal model, mice on high-fat, high-fructose, and cholesterol diets, compared to the control, showed LPS translocation and increased NASH severity [46,47]. LPS-triggered hepatic inflammation occurred through the activation of toll-like receptor 4 (TLR4) in several types of cells, including Kupffer cells, hepatocytes, hepatic stellate cells (HSCs), and liver sinusoidal endothelial cells (LSECs). In Kupffer cells, TLR4 signal activation via myeloid differentiation primary response 88 (MyD88) induced tumor necrosis factor (TNF)-α and reactive oxygen species (ROS), further enhancing hepatic inflammation. The LPS-triggered TLR4 on the HSCs induced the production of various chemokines and adhesion molecules, which in turn induced Kupffer cell chemotaxis. On the other hand, the activation of TLR4 on hepatocytes induced hepcidin production via the MyD88/c-Jun N-terminal kinase (JNK) pathway, which was associated with hepatic lipid accumulation [48]. These results were consistent with those of a previous human study that showed higher levels of antibodies against LPS, produced by Gram-negative bacteria, in NASH patients than in healthy individuals, and this increase paralleled disease severity [49]. In addition to LPS, other pathogen-associated molecular patterns (PAMPs), including peptidoglycan, flagellin, and bacterial RNA and DNA, can enter into the liver due to increased intestinal permeability and trigger inflammatory responses. TLR9 activation by bacterial DNA further induces IL-1β production in Kupffer cells, thus resulting in hepatic steatosis, inflammation, and fibrosis [50]. In addition to TLR, inflammasome proteins, which are activated by NLRs and assembled, recognize PAMPs, leading to IL-1 and IL-18 production and further triggering inflammation [51]. A previous study indicated that NLRP3 inflammasome components were significantly increased in NASH patients compared to in non-NASH NAFLD patients [52]. These results indicated the association between hepatic inflammation and NLRP3 inflammasome. Indeed, the lack of NLRP3 inflammasome attenuated hepatic injury, immune cell infiltration, and choline-deficient (CD) L-amino-defined (CDAA) diet-induced fibrosis, thereby confirming the important role of NLRP3 inflammasome [52]. The increased influx of different classes of lipotoxic lipids and insulin resistance-induced adipokines, in addition to PAMPs, into the liver due to a leaky gut can also trigger hepatic inflammation. Several lipid classes, including saturated non-esterified fatty acids (NEFAs), free cholesterol, sphingolipids, and sphingosine 1-phosphate (S1P), induce liver injury and inflammation [53,54]. For example, saturated NEFAs can bind to and activate hepatocyte death receptor TNF-related apoptosis-inducing ligand (TRAIL) receptor 2 (TRAIL-R2) and damage-associated molecular pattern (DAMP) receptors, such as TLR4, and further trigger downstream activation of the caspase cascade and execute hepatocyte apoptosis [55]. Additionally, accumulating ceramides, one type of sphingolipid, were observed in the HFD animals fed diets enriched with saturated fatty acids [56]. Increased ceramides contributed to ROS generation, and oxidative stress further induced apoptosis and inflammatory cell recruitment to the liver, thus resulting in worsening hepatic inflammation and damage [57]. Generally, lipotoxicity induces endoplasmic reticulum (ER) stress, mitochondrial dysfunction, inflammasome activation, and cell death [58] (Figure 3).

### 2.4. NASH and Gut Microbiota-Derived Metabolites

Dysbiosis includes changes in not only the gut microbiota composition but also in the microbiota-derived metabolites obtained from dietary nutrients, thereby affecting host metabolic homeostasis. The most important gut microbiota-derived metabolites are SCFAs. SCFAs are produced by the fermentation of dietary fibers by the gut microbiota, including *Roseburia*, *Ruminococcus*, *Salmonella*, *Blautia*, *Eubacterium*, *Anaerostipes*, *Coprococcus*, *Faecalibacterium*, *Marvinbryantia*, and *Megasphaera* spp. The relative abundance levels of acetate, propionate, and butyrate were the highest [59]. Propionate was associated with peptide-YY and glucagon-like peptide-1 (GLP-1) release using a primary cultured human colonic cell model and further demonstrated that increasing colonic propionate prevents weight gain and insulin resistance in overweight adult humans [60]. In an HFD-induced steatohepatitis mouse model, butyrate promoted CD4+ T cell differentiation into helper T 2 (Th2), Th22, or regulatory T (Treg) cells and inhibited CD4+ T cell differentiation into Th1 or Th17 cells, further preventing hepatic inflammation [61]. SCFAs exerted their biological functions mainly via G-protein coupled receptor (GPR) 41/43 activation or histone deacetylase (HDAC) inhibition. GPR41 and GPR43 were expressed in not only the gut but also the liver, and GPR41 and GPR43 activation attenuated host insulin resistance in murine models [62,63,64]. Butyrate could inhibit HDAC directly and regulate phosphorylation of cyclic adenosine monophosphate (cAMP) response element binding protein (CREB), which is involved in gluconeogenesis, further influencing the gut and liver metabolisms [65]. Thus, SCFAs affected not only the hepatic immune response but also hepatic metabolism.

Amino acid imbalance is often found in NAFLD patients [66,67]. The ratio of branched-chain amino acids (BCAAs) to aromatic amino acids (AAAs) is a diagnostic marker for the severity of liver dysfunction. A decreasing ratio indicates severe liver dysfunction. BCAAs, including valine, leucine, and isoleucine, are essential amino acids for human beings and are involved in liver disease pathophysiology. Cohort studies have indicated that serum BCAA levels are positively correlated with insulin resistance and steatosis [67,68,69]. Further studies showed that *Prevotella copri* and *Bacteroides vulgatus* were the main species responsible for the association between BCCA biosynthesis and insulin resistance, and this finding was confirmed in the mouse model [70]. Although several studies indicated that BCAAs could inhibit triglyceride (TG) deposition in hepatocytes, reduce ER stress, and enhance gut barrier function by improving immune response, some inconsistencies in the results indicated that BCAAs caused hepatic damage, associated with abnormal lipolysis, in mice on HFDs [71,72,73]. Phenylacetic acid (PAA) is an AAA-derived metabolite which is produced by the gut microbiota. PAA has been found to induce hepatic steatosis by lowering protein kinase B (Akt) phosphorylation and affect BCAA metabolism by increasing acyl-CoA dehydrogenase short/branched chain (ACADSB) expression in primary hepatocytes and mice, indicating the causal role of PAA in NAFLD [67].

Recent studies have indicated that tryptophan metabolites may affect NAFLD development [59]. Indole and its derivatives, including indoleacrylic acid (IA), indole-3-acetic acid (IAA), indole-3-aldehyde (I3A), indole-3-propionic acid (IPA), and tryptamine, are the main tryptophan-derived gut bacterial products which are mainly produced by *Bacteroides*, *Eubacterium*, *and Clostridium* spp. [59]. Among them, tryptamine and I3A reduced hepatic fatty acid synthase (FAS) and sterol regulatory element-binding protein-1c (SREBP1c) expression via aryl-hydrocarbon receptor (AhR), further reducing Kupffer cell-induced hepatic inflammation [74]. Lower abundance of IPA in obese patients has been reported in previous studies, and IPA supplementation resulted in the reduction of weight gain in the antibiotic-induced dysbiosis animal model [75]. Additionally, IPA improved intestinal barrier function via pregnane X receptor (PXR), which in turn inhibited endotoxin-induced TLR4 signaling and improved tissue inflammation [76,77]. Therefore, tryptophan metabolites appear to be potential therapeutic targets.

Higher levels of ethanol in the blood and breath, accompanied with up-regulation of hepatic alcohol dehydrogenase, aldehyde dehydrogenase, and CYP2E1, were exhibited in NASH patients and ob/ob mice without alcohol consumption [78]. These results indicated that endogenous ethanol might be involved in NAFLD pathogenesis. Endogenous ethanol is obtained by carbohydrate fermentation by gut microbiota, and it stimulates oxidative stress and aggravates liver inflammation in NAFLD [79,80]. *Escherichia coli*, *Enterobacteriaceae* spp., and *Klebsiella pneumonia* have been identified as ethanol-producing bacteria and were found to be relatively abundant in NAFLD patients [79,81]. Ethanol can be further metabolized into acetaldehyde, which induces hepatic injury [82]. Therefore, increasing endogenous ethanol production may deteriorate hepatic inflammation. Ethanol exerts direct toxic effects on the liver, increasing intestinal permeability, which results in endotoxemia, and further triggers the inflammatory response, contributing to liver injury [83]. These findings indicate that endogenous ethanol might play a pivotal role in NASH pathogenesis. However, further investigation is required to determine the exact effects of endogenous ethanol on NAFLD and NASH.

BAs can be divided into primary and secondary BAs. Primary BAs, including cholic acid (CA) and chenodeoxycholic acid (CDCA), are produced using cholesterol in the liver. Primary BAs are converted into more than 20 secondary BAs, including deoxycholic acid (DCA) and lithocholic acid (LCA), by the gut microbiota [84,85]. Furthermore, distinct BA profiles were observed between the germ-free and conventional animals, thereby indicating the direct effects of the gut microbiota on BAs [86]. Adams et al. (2020) showed that increased DCA was associated with not only the increased relative abundance of specific bacterial groups, including *Bacteroidaceae* and *Lachnospiraceae* spp., but also advanced fibrosis in NAFLD [87]. At the molecular level, individual BAs act as agonists or antagonists for farnesoid X receptor (FXR) and takeda G-protein-coupled bile acid receptor 5 (TGR5) and affect energy, glucose, and lipoprotein metabolism, indicating that altered BA composition may affect host metabolism by modifying these signals [88]. For example, FXR activation by CA, CDCA, and obeticholic acid (OCA), which is derived from CDCA, stimulated production of fibroblast growth factor 15 (FGF15) in mice or FGF19 in humans. FGF15 and FGF19 further bound to FGF14 in the liver to inhibit BA synthesis, thereby altering the BA pool and exhibiting NASH improvement [89,90,91,92]. On the other hand, increasing CA or DCA may result in dysbiosis owing to their anti-microbial activity, further contributing to NAFLD pathogenesis [93,94]. Additionally, TGR5 activation in the intestine results in GLP-1 release from L cells, further promoting insulin release from pancreatic β–cells [95,96]. Taken together, these results indicate that the gut microbiota-induced alteration of the BA pool plays an important role in NAFLD pathogenesis.

**Figure 4:**
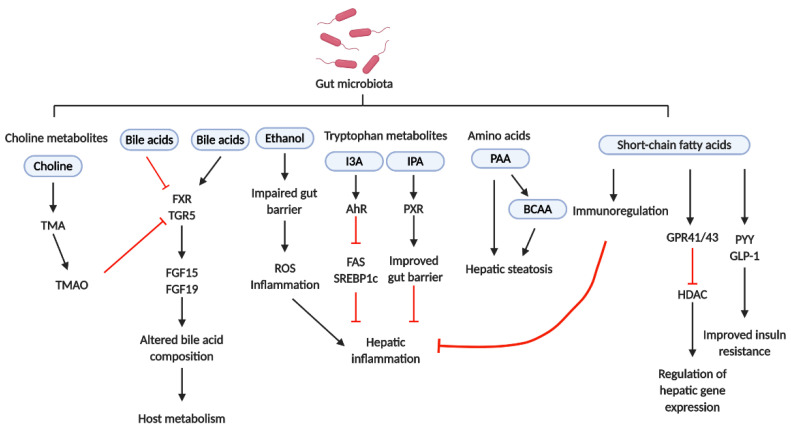
**The roles of gut microbiota-derived metabolites.** Short-chain fatty acids (SCFAs) are associated with the release of PYY and GLP-1, which further ameliorate insulin resistance. SCFAs also activate GPR41/43, performing the regulation of hepatic gene expression via inhibition of HDAC. Additionally, SCFAs also perform immunoregulation to inhibit hepatic inflammation. PAA can induce hepatic steatosis by itself or via affecting BCAA metabolism. IPA improves intestinal barrier function via PXR, which improves tissue inflammation. I3A reduces hepatic FAS and SREBP1 expression via AhR, further reducing hepatic inflammation. Increasing endogenous ethanol production may deteriorate hepatic inflammation. Specific BAs act as agonists or antagonists of FXR and TGR5 which affect the composition of BAs and further affect host metabolism. TMAO, which is derived from TMA and choline, modulates BA metabolism and FXR signaling inhibition, contributing to NAFLD pathogenesis. TMA, trimethylamine; TMAO, trimethylamine-N-oxide; FXR, farnesoid X receptor; TGR5, Takeda G protein-coupled bile acid receptor 5; FGF, fibroblast growth factor; I3A, indole-3-aldehyde; IPA, indole-3-propionic acid; AhR, aryl-hydrocarbon receptor; PXR, pregnane X receptor; FAS, fatty acid synthase; SREBP1c, sterol regulatory element-binding protein-1c; PAA, phenylacetic acid; BCAAs, branched-chain amino acids; GPR, G-protein coupled receptor; GLP-1, glucagon-like peptide-1; PYY, peptide-YY; HDAC, histone deacetylase.

Choline is metabolized to phosphatidylcholine, which is essential for very low-density lipoprotein (VLDL) production and hepatic lipid transfer, in the liver. Most phosphatidylcholine is derived from dietary choline. Therefore, hepatic lipid metabolism is affected by choline deficiency. CD diets are commonly used to induce NAFLD in animal models. Decreased VLDL levels and β-oxidation were observed in mice on CD diets, further resulting in fatty acid and cholesterol accumulation and increased oxidative stress and inflammation in the liver [20,97]. Three major bacterial phyla, including Proteobacteria, Firmicutes, and Actinobacteria, are associated with choline metabolism. These bacteria metabolize dietary choline to trimethylamine (TMA), which is further metabolized to trimethylamine-N-oxide (TMAO) by flavin-containing monooxygenases, in the liver [98]. High circulating TMAO levels have been reported to increase the risk of atherosclerosis and cardiovascular disease [99]. Previous studies also indicated that NAFLD patients exhibited elevated serum TMAO levels, which were positively correlated with the pathological progression of NAFLD [100]. Further research indicated that TMAO modulated BA metabolism and FXR signaling inhibition, contributing to NAFLD pathogenesis [101]. The effects of the gut microbiota-derived metabolites on NAFLD are shown in Figure 4 and Table 1.

## 3. From NASH to HCC: The Gut Microbiota-Associated Mechanisms

**Figure 5:**
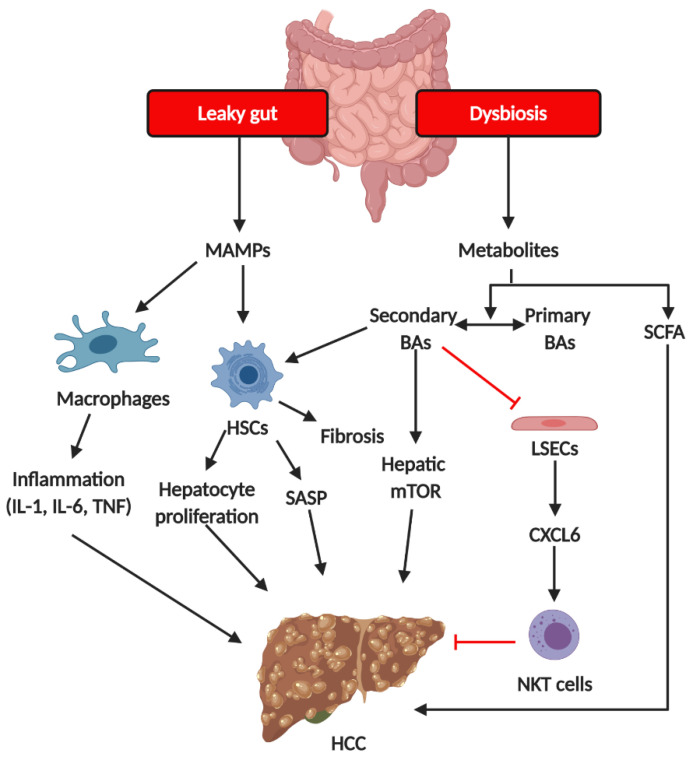
**The mechanism of gut microbiota on the pathogenesis of HCC.** Increased hepatic exposure to microbiota-derived metabolites and MAMPs results from dysbiosis and a leaky gut. Changes in BA pool (the ratio of primary BAs and secondary BAs) alter LSEC- and CXCL16-dependent NKT recruitment as well as HSC SASP. MAMPs induce the activation of macrophages, resulting in the production of pro-inflammatory cytokines including IL-1, IL-6, and TNF. Increased pro-inflammatory cytokines further contribute hepatic inflammation, which may also promote HCC development. MAMPs, microbiota-associated molecular patterns; HSCs, hepatic stellate cells; SASP, senescence-associated secretory phenotype; BAs, bile acids; LSECs, liver sinusoidal cells; SCFAs, short chain fatty acids; NKT cells, nature killer cells.

NASH progression to HCC shows mechanisms similar to those of NAFL progression to NASH. Dysbiosis and a leaky gut result in PAMP and gut microbiota-derived metabolite influx into the liver, thereby further triggering hepatic inflammation and disrupting metabolism homeostasis. Several groups of bacteria were associated with HCC. A previous study showed *E. coli* overgrowth in the intestines of HCC and cirrhosis patients [102]. Another study indicated that HCC patients, compared to cirrhosis patients, exhibited increased levels of *Bacteroides* and *Ruminococcaceae* spp. [103,104] and decreased levels of *Akkermensia* and *Bifidobacterium* spp. [104]. At the molecular level, PAMPs, such as LPS, activated signaling of TLRs, including TLR4 and TLR9, and induced cytokine and chemokine production, further inducing immune cell infiltration into the liver. PAMPs also activated HSCs via TLR activation to senescence-associated secretory phenotype (SASP) and induced epiregulin production, further promoting fibrosis development [105,106] (Figure 5). Dysbiosis affects metabolic functions via the gut microbiota-derived metabolites in NASH progression to HCC, like in NAFL progression to NASH. Primary BA conversion to secondary BAs by the gut microbiota is involved in HCC pathogenesis. Dysbiosis promotes HCC by inhibiting primary BA production, further inhibiting LSEC activation. Inhibition of LSEC activation results in chemokine ligand 6 (CXCL6) down-regulation, CXCL6-mediated natural killer T cell recruitment, and further loss of liver tumor growth control [107]. On the other hand, secondary BAs promote HCC development by activating HSC SASP and the hepatic mTOR pathway [108]. Thus, controlling the production of secondary BAs using antibiotics reduces HCC development [109] (Figure 5).

## 4. Potential Therapeutic Strategies and Non-Invasive Diagnosis

Owing to the higher daily calorie intake and sedentary lifestyle of NAFLD patients, the first step of NAFLD treatment includes weight loss by lifestyle modifications, including diet restriction and increased physical activity [110]. However, hepatic fat accumulation, inflammation, and necrosis are significantly improved only when more than 10% of the body weight is reduced [111,112]. Thus, lifestyle therapy appears to be insufficient for resolving NASH.

In addition to lifestyle interventions, potential NAFLD therapeutic strategies based on the gut microbiota and gut-liver axis have attracted attention in recent years. Antibiotic, prebiotic, and probiotic use can be applied to modulate the gut microbiota and prevent hepatocarcinogenesis. For example, a preclinical mouse model indicated that chronic oral administration of antibiotics decreased secondary bile acid levels, hepatic lipid accumulation, and attenuated hepatic inflammation and fibrosis via modulating the composition of gut microbiota [113,114]. On the contrary, Mahana D. et al. showed different results which indicated that mice treated with antibiotics exhibited severe insulin resistance and NAFLD and the composition of the gut microbiota was shifted from Firmicutes to Bifidobacterium, S24-7, and Prevotella [115]. The function of the gut microbiota is based on community, and a “healthy” microbiome has not been defined yet [116]. Therefore, these inconsistent results may arise from the complex community of gut microbiota. Otherwise, antibiotics may eliminate important species associated with healthy status and the risk of antibiotic resistance poses a larger concern, thereby reducing the efficiency of antibiotic use as a therapeutic strategy. Food ingredients which improve beneficial bacterial growth in the gut are termed prebiotics. In humans, supplementation with prebiotics such as oligofructose decreases the levels of hepatic inflammatory markers [117]. A previous study indicated that prebiotic treatment was negatively associated with endotoxin levels [118]. In addition to human studies, several animal studies have revealed the therapeutic potential of prebiotics. For example, prebiotic treatment reduced hepatic TG accumulation via the inhibition of expression of genes such as FAS, which is involved in the lipogenesis pathway [114]. On the other hand, probiotics are live bacteria which are beneficial to the host. For example, *Lactobacillus* and *Bifidobacterium* spp. have been reported to reduce gut inflammation and improve gut barrier function by remodeling the gut microbiota [119]. In humans, administration of *Lactobacillus acidophilus* reduced AST and ALT levels in NASH patients [120] and several clinical trials of probiotics were reported in other reviews [121]. However, most of the molecular mechanisms by which probiotics exert their functions remain unclear.

FMT is a novel therapeutic strategy which is defined as the transplantation of functional gut microbiota in healthy human feces into patients to alter the recipient’s gut microbiota directly and normalize microbiota composition for therapeutic benefit [122]. Remarkable effectiveness of FMT was shown in patients with recurrent and refractory *Clostridium difficile* infection and has been confirmed as a clinical technique for treatment according to the 2013 guidelines [123,124,125]. FMT application, as a treatment strategy for extra-gastrointestinal diseases, has been evaluated in recent years. A previous study indicated that mice on HFDs showed decreased hepatic lipid accumulation and pro-inflammatory cytokine levels after FMT [126]. Additionally, FMT elevated the relative abundance of the beneficial bacterial species of *Christensenellaceae* and *Lactobacillus*, improved gut barrier function, and increased butyrate production, thereby further ameliorating endotoxemia [126]. In a human study, FMT from lean donors to individuals with metabolic syndrome temporarily increases insulin sensitivity [127]. A phase I clinical study has indicated that FMT with oral capsule restores microbial diversity and function and further reduced the recurrence of hepatic encephalopathy [128,129]. Although these human studies were not targeted toward the therapy of NAFLD, these findings still indicate the therapeutic potential of FMT. However, only a few control trials of FMT have been enrolled to date, and the role of FMT must be further examined because FMT application involves the risk of developing other pathogenic infections [130].

In addition to potential treatments, effective and non-invasive methods for diagnosing NAFLD are another important key to preventing HCC. Unfortunately, broadly applicable and non-invasive methods for diagnosing NAFLD are not available as yet. A recent study by Oh T.G. et al. demonstrated that a core gut microbiome signature can identify cirrhosis across separated cohorts, independent of disease etiology, host genetic, and environmental factors [131]. The identified disease microbiome included the elevated relative abundance of *Veillonella parvula*, *Veillonella atypica*, *Ruminococcus gnavus*, *Clostridium bolteae*, and *Acidaminococcus* sp. D21 and decreased abundance of *Eubacterium eligens*, *Eubacterium rectale*, and *Faecalibacterium prausnitzii* [131]. Although the results indicated the improved diagnostic accuracy in several cohorts, the authors claimed that these diagnostic methods need multi-center studies and well-phenotyped patients in order to be validated. However, it still is a promising non-invasive diagnostic method for NAFLD.

## 5. Conclusions

In general, the current NAFLD therapeutic strategies based on the gut microbiota and gut–liver axis mainly include prebiotic, probiotic, and FMT application. These therapeutic strategies improve NAFLD and HCC by recovering gut homeostasis from a state of dysbiosis, thereby improving gut barrier function to prevent endotoxemia, promoting anti-inflammatory effects and modulating gut microbiota-derived metabolite production. However, a huge gap in the development of therapies by targeting specific gut microbiota species or gut microbiota-derived metabolites remains. Although high-throughput sequencing including 16S rRNA and metagenomic sequencing help the researcher to identify gut microbiotas that are present in a sample without the need for culturing, the results only indicate the correlation of gut microbiota with diseases. Moving from association to causation remains a significant challenge. Specific species of gut microbiota may need to be cultured in order to conduct the causation test. Therefore, there is a strong demand for a culturomic technique. On the other hand, due to the complex community of gut microbiota, multi-omics analysis including transcriptomics, proteomics, and metabolomics may give us a glimpse of the entire disease picture and further contribute to the development of precision medicine. Therefore, advances in the understanding of the gut microbiota will allow the development of improved diagnostic, prognostic, and therapeutic strategies for liver diseases.

## Figures and Tables

**Table 1:** Overview of metabolite-relative effects on host in in vitro and animal models.

**Metabolites**	**Effects**	**References**
Acetate	HDAC inhibition.	[65]
Propionate	Induces PYY and GLP-1 release. HDAC inhibition.	[60,65]
Butyrate	Promotes Th2, Th22, or Treg cell differentiation, further preventing hepatic inflammation. HDAC inhibition.	[61,65]
PAA	Induces steatosis.	[67]
BCAA	Alleviates hepatic steatosis and liver injury by suppressing FAS gene expression and protein levels. Suppresses the progression of NASH by reducing oxidative stress.	[71,72]
	Exacerbates hepatic oxidative stress, increases hepatic apoptosis.	[73]
Tryptamine	Reduces production of pro-inflammatory cytokines and migration of macrophages.	[74]
I3A	Reduces production of pro-inflammatory cytokines and migration of macrophages. Reduces the expression of FAS and SREBP1c.	[74]
IPA	Reduces weight gain. Improves intestinal barrier function.	[75,76]
Ethanol	Transfer of high-alcohol-producing Klebsiella pneumoniae by oral gavage into mice induces NAFLD.	[81]
Obeticholic acid (OCA)	Decreases hepatic inflammation by inhibition of pro-inflammatory cytokines. Decreases fibrogenesis by inhibition of pro-fibrotic cytokines. Inhibits LSEC and Kupffer cell activation.	[92]
Cholic acid (CA) Deoxycholic acid(DCA)	Changes in the composition of gut microbiota.	[94]
TMAO	Increases hepatic TG accumulation and lipogenesis. Shifts hepatic BA composition toward FXR-antagonistic activity.	[101]
**Human Studies**		
**Metabolites**	**Effects**	**References**
Propionate	Prevents weight gain and insulin resistance.	[60]
BCAA	Positive correlation with insulin resistance and steatosis.	[67,68,69]
IPA	Negative correlation with obesity.	[75,76]
Ethanol	Positive correlation with NASH.	[79]
DCA	Associated with fibrosis in NAFLD.	[87]
OCA	Reduction in ALP, ALT and GGT.	[91]
TMAO	Positively correlated with NAFLD. Positively correlated with the serum levels of total BA and hepatic CYP7A1 mRNA.	[100,101]

## Abbreviations

HCC	hepatocellular carcinoma
NAFLD	non-alcoholic fatty liver disease
NASH	non-alcoholic steatohepatitis
HBV	hepatitis B viruses
HCV	hepatitis C viruses
SCFA	short chain fatty acid
ZO-1	zonula occludens-1
DSS	dextran sodium sulfate
IL10	interleukin-10
IL6	interleukin-6
PPAR	peroxisome proliferator-activated receptor
FMT	fecal microbial transplantation
HFDs	high-fat diets
NLRP6	nucleotide-binding oligomerization domain -like receptor family pyrin domain containing 6
ASC	apoptosis-associated speck-like protein containing a caspase recruitment domain
TLR4	toll-like receptor 4
HSCs	hepatic stellate cells
LSECs	liver sinusoidal endothelial cells
MyD88	myeloid differentiation primary response 88
TNF-α	tumor necrosis factor-α
ROS	reactive oxygen species
JNK	c-Jun N-terminal kinase
PAMPs	pathogen-associated molecular patterns
NEFAs	non-esterified fatty acids
S1P	sphingosine 1-phosphate
TRAIL	TNF-related apoptosis-inducing ligand
DAMP	damage-associated molecular pattern
GPR	G-protein coupled receptor
HDAC	histone deacetylase
CREB	cyclic adenosine monophosphate (cAMP) response element binding protein
BCAAs	branched-chain amino acids
AAAs	aromatic amino acids
ACADSB	acyl-CoA dehydrogenase short/branched chain
IA	indoleacrylic acid
IAA	indole-3-acetic acid
I3A	indole-3-aldehyde
IPA	indole-3-propionic acid
SREBP1c	sterol regulatory element-binding protein-1c
AhR	aryl-hydrocarbon receptor
PXR	pregnane X receptor
CA	cholic acid
CDCA	chenodeoxycholic acid
DCA	deoxycholic acid
LCA	lithocholic acid
FXR	farnesoid X receptor
TGR5	takeda G-protein-coupled bile acid receptor 5
OCA	obeticholic acid
FGF	fibroblast growth factor
VLDL	very low-density lipoprotein
TMA	trimethylamine
TMAO	trimethylamine-N-oxide
CXCL6	chemokine ligand 6
